# Word reading threshold and mild cognitive impairment: a validation study

**DOI:** 10.1186/1471-2318-12-38

**Published:** 2012-07-24

**Authors:** Genevieve Arsenault-Lapierre, Howard Bergman, Howard Chertkow

**Affiliations:** 1 Bloomfield Center for Research on Aging, Lady Davis Institute in Medical Research, Sir Mortimer B. Davis Jewish General Hospital, Montreal, Canada; 2 Department of Geriatric Medicine, McGill University, Montreal, Canada; 3 Division of Geriatric Medicine, Department of Medicine, Sir Mortimer B. Davis Jewish General Hospital, Montreal, Canada; 4 Department of Clinical Neurosciences, Sir Mortimer B. Davis, Jewish General Hospital, Montreal, Canada; 5 Lady Davis Institute, 3755 Chemin de la Côte Ste-Catherine, Montréal, Québec, H3T 1E2, Canada

## Abstract

**Background:**

It was previously found, in a pilot study, that Word Reading Threshold (WRT) test is abnormally prolonged in individuals with Alzheimer’s disease (AD) and Mild Cognitive Impairment (MCI), with high sensitivity and specificity. This validation study examines the WRT test as a prognostic tool in MCI individuals. We wish to confirm in a larger group the sensitivity and specificity of the WRT test and determine whether it is influenced by deterioration on other cognitive domains.

**Methods:**

We measured WRT in 60 MCI individuals, 29 AD patients, and 33 normal elderly control (NE). We followed the MCI individuals over 8 years to monitor who progressed to dementia.

**Results:**

We found a statistically significant difference in WRT scores between the three groups. However, using the same cutoff of 85 milliseconds suggested by Massoud and his colleagues, we found lower diagnostic sensitivity (72%) and specificity (76%) when comparing NC and AD. Furthermore, the test did not clearly differentiate MCI individuals who progressed to dementia from those who did not. WRT was found to correlate to some degree with other cognitive domains, especially attention.

**Conclusions:**

We conclude that the WRT is insufficient alone as a diagnostic tool for prodromal AD.

## Background

The term Mild Cognitive Impairment (MCI) describes a group of individuals who are at an intermediate stage between normal aging and dementia from clinical, neurological, and neuropsychological standpoints. In fact, individuals with MCI present with subjective memory complaints, corroborated by objective deficits on mental status testing and standardized neuropsychological tests, without functional or social impairment needed to diagnose dementia [1]. Prospective studies show that these individuals are at an increased risk of developing AD. Indeed, 10% to 15% of the individuals with MCI will progress to dementia each year, compared to an annual rate of 1% to 2% in the general population over 65 years old [1]. Ultimately, in most memory clinics, between 50% and 75% of patients presenting with MCI will go on to dementia over long term follow-up [2,3]. MCI individuals often show hippocampal atrophy, a characteristic feature of Alzheimer’s disease (AD), to a degree midway between that of normal individuals and demented patients [4]. Recently, it has even been proposed that MCI individuals with MRI atrophy or other AD-like biomarkers, be considered as having prodromal AD [5].

The existence of such a group of individuals at risk of developing AD has encouraged research on diagnostic tools that could discriminate those who will progress to AD. Because of the demands of clinical settings, such discriminative tests ought to be relatively inexpensive, fairly quick to administer and opaque to other factors that can affect cognitive functions such as depression [6,7]. Memory loss being the core symptom of AD, research has focused largely on neuropsychological tests that assess this symptom. However, memory loss can be easily confounded with encoding and attention deficits seen in other conditions such as depression [8-10]. More sophisticated memory tests may need to be administered by neuropsychologists. At the same time, more studies show that episodic memory deficits might not be the only feature of mild AD [11]. For example, studies have found subtle visuoperceptual deficits in AD, particularly changes in word perceptual threshold [7]. Perceptual tests, being for less attention demanding, are likely to be less influenced by depression – a major advantage for an AD tests. One explanation of the deficits observed in word-recognition tests could be the degeneration of the “word-form area” in the occipital-temporal extrastriate cortex [12] also affected, albeit to a lesser degree than memory areas, in AD [13,14]. Another possible explanation could be that patients with AD show poorer performance on more demanding perceptual tasks because they have slower central processing abilities. For instance, a masked word recognition test is more demanding on the central processing abilities than an unmasked letter recognition test, and therefore, the former test would be more sensitive in the earlier disease stages.

Based on these premises, our group conducted a pilot study [15], testing the discriminative power of the Word Reading Threshold (WRT) test. In brief, we used a computerized test designed to assess visual perception changes encountered in AD or MCI. We found that the mean WRT was significantly longer in the 13 patients with AD than in the 12 normal controls (NC), and, with a threshold of 85 milliseconds (ms), there was a diagnostic sensitivity of 77% and a diagnostic specificity of 92%. It was also found that all of the four individuals with MCI who progressed to dementia after two years of follow-up also scored above that threshold. This test is inexpensive and simple to administer. These results therefore warranted confirmation in a larger group of subjects in order to test the specificity, reliability, and replicability of WRT, as well as its opacity to other cognitive domains. Consequently, the goal of this study is to validate the WRT as a diagnostic tool in MCI individuals. More precisely, we want to measure its sensitivity and specificity in a larger sample of AD, MCI and NC individuals, and examine potential cognitive correlates of word recognition and prolonged WRT.

## Methods

MCI and AD subjects were recruited from the memory clinic of the Jewish General Hospital, a tertiary care referral centre at McGill University in Montreal, Canada. The diagnostic criteria for AD were those suggested by NINCDS-ADRDA [16]. The general criteria for a diagnosis of MCI utilized were 1) subjective memory complaints, 2) corroborated by objective impairment on neuropsychological tests compared to age and education matched controls, 3) no functional or social impairment, 4) cognitive and functional impairment not sufficient to meet criteria for dementia [17]. As part of the usual evaluation at the clinic, each of the MCI and AD subjects underwent a thorough physical and neurological examination to rule out other reversible causes of cognitive decline, as well as extensive neuropsychological testing. Normal elderly controls (NC) were recruited from the Family Medicine Clinic of the Jewish General Hospital, from advertisements in newspapers or conferences on memory and from relatives of the MCI and AD subjects. They were screened to exclude those with memory complaints, and they underwent the same neuropsychological examination as the other two groups. MCI individuals were followed at the memory clinic about once a year for over 8 years to monitor those who progressed (MCIp) and who did not progress (MCInp). Table 1 summarizes the subjects’ demographics. All subjects signed a consent form and the study was in compliance with the Helsinki Declaration and approved by the Jewish General Hospital Research Ethic Board.

**Table 1:** Demographic and neuropsychological information

	**NC**		**MCI**		**AD**		
	**Mean**	**(SD)**	**Mean**	**(SD)**	**Mean**	**(SD)**	**AUC**
N	33		60		29		122
EDU^a,^	13,8	(2,90)	11,2	(3,1)	11,9	(3,7)	-
AGE^a, b^	74,7	(6,00)	75,0	(7,0)	78,1	(7,8)	-
GDS^a, b, c^	3,4	(4,20)	6,7	(4,9)	7,4	(5,4)	-
Duration	-	-	3,9	(3,2)	4,0	(2,2)	-
MMSE^a, b, c^	29,0	(1,10)	27,1	(2,2)	22,9	(3,9)	0,48
**WRT**^**a, b, c**^	**58,2**	**(24,10)**	**73,4**	**(25,7)**	**92,6**	**(31,7)**	**0,45**
*Memory*							
LM2^a, b, c^	12,7	(3,50)	5,8	(4,0)	1,5	(2,3)	0,82*
VR2 ^a, b, c^	49,8	(19,30)	20,6	(17,5)	3,8	(7,5)	0,71
RAVLT delay ^a, b, c^	8,6	(2,90)	3,2	(2,9)	0,4	(0,9)	0,76*
*Language*							
COWA lexical^a, b^	49,0	(16,30)	32,8	(12,9)	25,8	(14,8)	0,51
COWA semantic ^a, b, c^	17,9	(5,50)	13,0	(4,4)	7,4	(4,1)	0,48
BNT ^a, b, c^	53,7	(7,00)	47,0	(8,7)	38,3	(11,0)	0,46
*Attention*							
TRAIL ^a, b, c^	41,5	(12,30)	52,8	(20,4)	68,3	(29,0)	0,45
STROOPd^a, b^	14,7	(3,50)	16,9	(4,7)	24,4	(14,8)	0,49
STROOPw^a, b^	19,0	(4,70)	23,0	(5,8)	36,6	(33,8)	0,48
STROOPc^a, b^	30,9	(6,80)	41,7	(11,4)	69,0	(44,2)	0,63

Numbers represent means (standard deviation). Age and EDU; education, are measured in number of years. NC stands for normal elderly controls; MCI, Mild Cognitive Impairment; AD, Alzheiemer Disease; AUC, Area Under the Curve from ROC analyses on MCInp vs MCIp; GDS, Geriatric depression Scale; WRT, Word Reading Threshold is in milliseconds; MMSE, Mini-Mental Status Examination; LM2, Logical Memory delayed; VR2, Visual Reproduction delayed; RAVLT delayed, Rey-Auditory Verbal Learning Test delayed; TRAIL, Trail Making Test Subscale A; STROOPd, dots subscale of Stroop test; STROOPw, words subscale of Stroop Test; STROOPc, colors subscale of Stroop Test. ^a^, indicates significant difference between NC and MCI; ^b^, indicates significant difference between NC and AD, ^c^, indicates significant difference between MCI and AD and * indicates significant AUC difference between MCInp and MCIp groups (p < 0.002).

**Figure 1:**
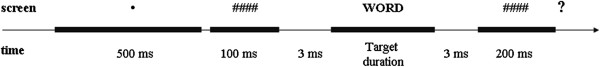
**Presentation of stimuli in word teading threshold test.** Above the arrow line is what is seen by the participant, and below is the actual time presentation of the items presented. Briefly, on a computer screen, a dot announcing the presentation of a noun appears for 500 milliseconds (ms), and is immediately followed by a mask, consisting of dash signs of same height and length as target noun, for 100 ms. Nouns are presented at increasing duration. After a 3 ms time lapse, a backward mask, consisting of the same dash signs is presented for 200 ms, followed by a question mark prompting a response from the participant. The target duration at which 5 out of 10 nouns are correctly read is the threshold for word recognition [15].

Detailed procedures for the WRT assessment are described in Massoud et al. [15]. In brief, four to six letters nouns are presented on a computer screen, matched across blocks for frequency. These nouns are presented in blocks of ten words, each block with an increasing presentation time. Forward and backward masking consists of a series of number signs of the same size and length as the target words. Subjects are asked to read each word out loud. The percentage of the words in a block that were correctly read is calculated. The WRT score is defined as the presentation duration at which 50% of the words in a block are read correctly. Figure 1 depicts the presentation of the stimuli on the screen.

To calculate the sensitivity and the specificity of the WRT at the suggested cut-off score of 85 ms, we compared NC and AD patients as follow. Sensitivity is TP/(TP + FN) and Specificity is TN/(TN + FP), where TP is the number of true positives; FP, the number of false positives; FN, the number of false negatives; and TN, the number of true negatives. We also carried a Receiver-Operating Characteristic (ROC) analysis to evaluate the performance of WRT in classifying appropriately NC and AD, and MCIp and MCInp.

Finally, to verify whether the WRT is opaque to other cognitive domains, we correlated the WRT scores with tests of memory, language and attention. We tested the subjects on Logical Memory (LM) I and II subscales and Visual Reproduction (VR) I and II subscales of the Weschler Memory Scale-Revised (WMS-R [18]), Rey Auditory-Verbal Learning Test (RAVLT [19]), Boston Naming Test (BNT [20]), Controlled Word Association Test (COWA [21]), the Trail Making Test, subscale A (TRAIL [22]), and Stroop Test [23].

To determine group differences in WRT scores, one-way ANOVA were performed, controlling for confounding variables, such as the number of years of education and depression scores on the Geriatric Depression Scale (GDS). To specify which pair-groups were different, Bonferroni’s post hoc analyses were performed. A difference was deemed statistically significant if the p-value was smaller than 0.05. ROC analyses were performed on WRT and other neuropsychological tests comparing NC and AD first, and then comparing MCIp and MCInp. To assess the opacity of WRT to other cognitive functions, performances on WRT were correlated with neuropsychological tests using partial correlations to control for confounding variables (education and depression), in the whole study sample first, then in each diagnostic group. Bonferroni’s corrections for multiple comparisons were applied to the partial correlations and a p-value of 0.005 was used.

## Results

We collected WRT scores for 60 individuals with MCI, 29 patients with AD, and 33 NC. The three groups differed in terms of years of education (F_(2,120)_ = 6.9, p = 0.001) and depression scores as measured by Geriatric Depression Scale (GDS [24]: F_(2,111)_ = 6.0, p = 0.003), which correlated with WRT scores. Therefore, education and GDS were entered as covariates in our analyses. Age difference did not reach statistical significance.

**Figure 2:**
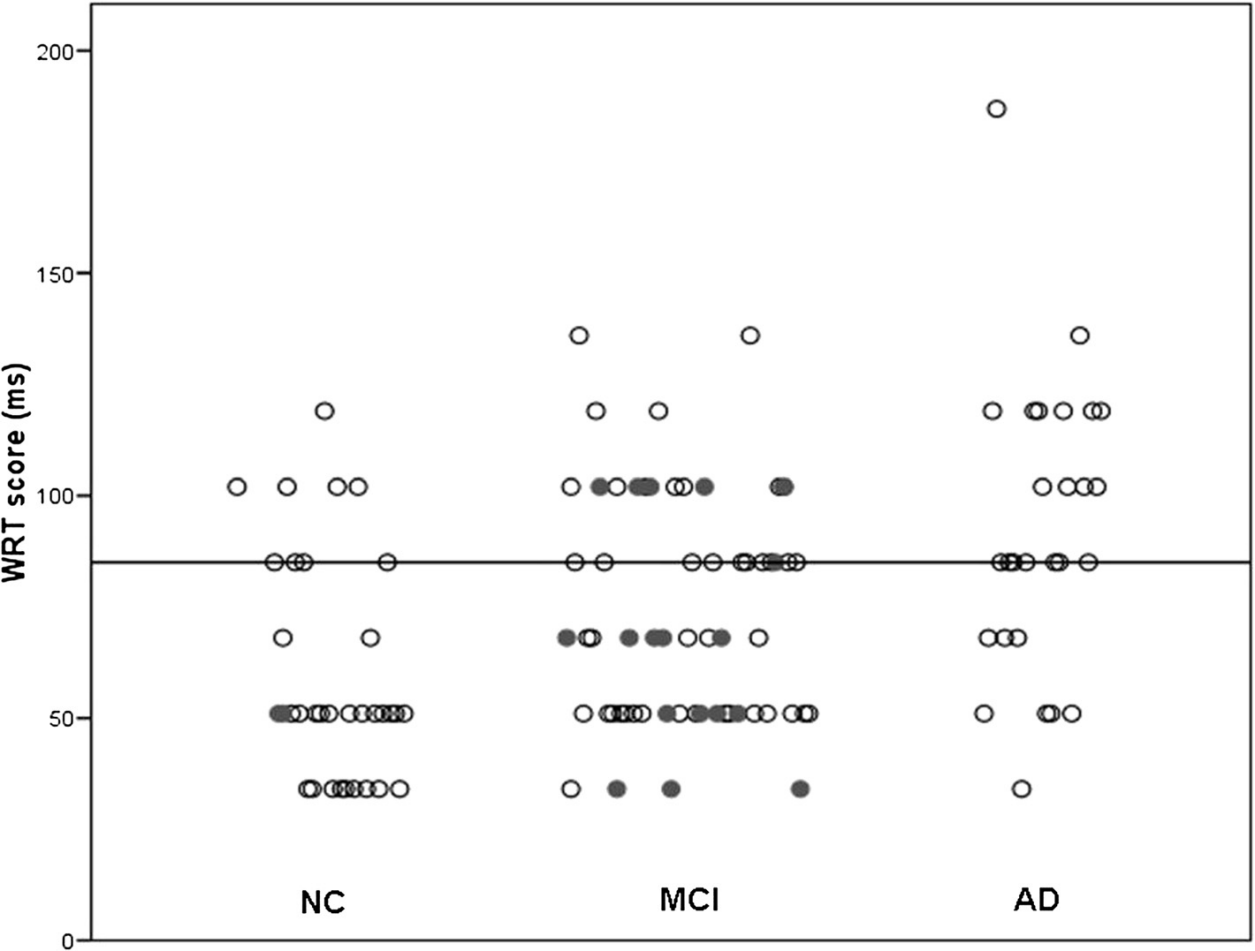
**Individual word reading threshold scores in the three groups.** Individual Word Reading Threshold (WRT) scores expressed in milliseconds (ms) in normal elderly controls **(NC)**, individuals with Mild Cognitive Impairment **(MCI)**, and patients with Alzheimer’s disease **(AD)**. Dark circles indicate individuals who had progressed to AD or MCI at 8 years follow-up. Bar represents the cut-off proposed by Massoud et al. (2002) above which scores should indicate potential progression from **MCI** to **AD.**

**Figure 3:**
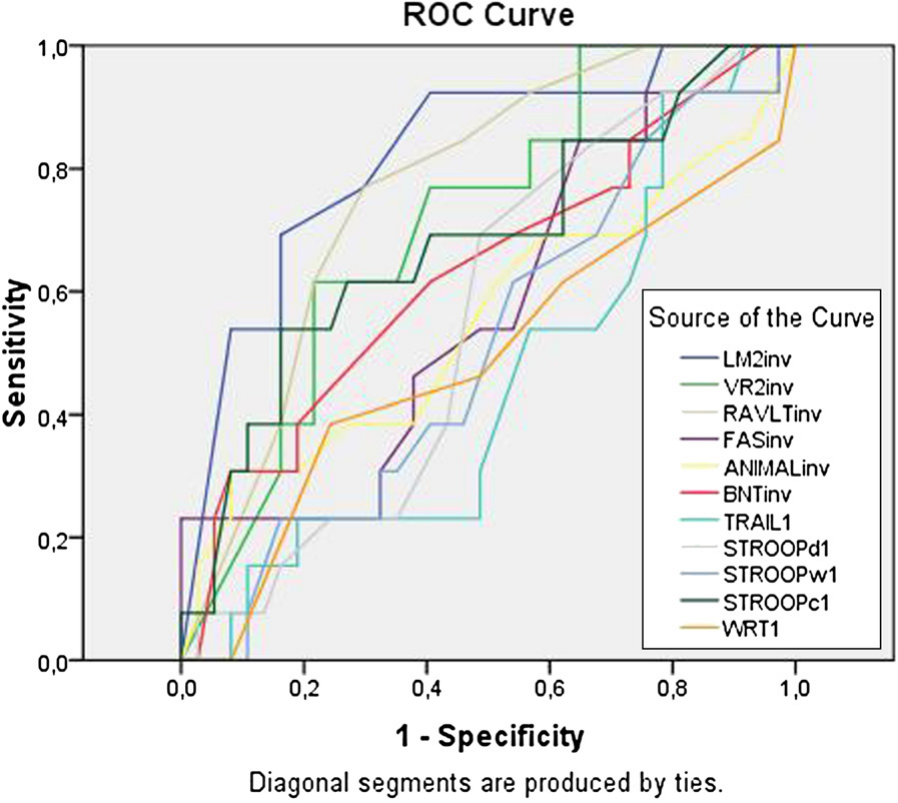
**ROC curves for individual tests in differentiating MCI progressors from MCI non-progressors.** Lines represent individual ROC curves for individual tests in differentiating MCI who progressed to AD from those who did not. WRT stands for Word Reading Threshold is in milliseconds; LM2, Logical Memory delayed; VR2, Visual Reproduction delayed; RAVLT delayed, Rey-Auditory Verbal Learning Test delayed; COWA, Controlled-Word Association; BNT, Boston Naming Test; TRAIL, Trail Making Test Subscale A; STROOPd, dots subscale of Stroop test; STROOPw, words subscale of Stroop Test; STROOPc, colors subscale of Stroop Test.

We found significant differences in the groups’ performance on WRT (F_(2,119)_ = 12.7, p < 0.01). Bonferroni’s post-hoc analyses revealed that significant differences existed between the three groups (p < 0.03). Figure 2 displays individual WRT scores in each group. Using the cut-off determined in Massoud’s study (85 ms), we found a diagnostic sensitivity of 72% and a specificity of 76%. ROC analyses showed that WRT differentiate between NC and AD about 81% of the time (p < 0.001). However, the test does not perform better than chance alone at differentiating MCIp and MCInp (Area Under the Curve, AUC = 45%, p = 0.51). Indeed, WRT test correctly classified 6 out of the 19 MCI who had progressed to AD after 8 years of follow-up. Logical Memory (LM) delayed and Ray Auditory Verbal Learning Test (RAVLT) delayed, both tests assessing delayed verbal memory, were better at discriminating between MCI subjects who progressed (MCIp) and those who did not (MCInp). The AUC of ROC analyses comparing the neuropsychological tests between MCIp and MCInp are represented in Table 1 and Figure 3.

We found significant correlations, after Bonferroni’s corrections for multiple comparisons, with memory as measured with Visual Reproduction (VR) II (r = −0.32, p = 0.003) and RAVLT delayed (r = −0.35, p = 0.001). Scores on WRT also correlated with language tests scores, as measured by Boston Naming Test (BNT; r = −0.32, p = 0.002), Controlled Word Association (COWA) lexical subtest (r = −0.35, p = 0.001) and semantic subtest (r = −0.34, p = 0.001). However, these correlations might have been spurious, as they did not remain statistically significant when the correlations were performed in the diagnostic groups independently, with the exception of COWA lexical subtest in the NC (p = 0.002). WRT was found to correlate with tests of attention, such as the Trail Making subscale A (TRAIL; r = 0.48, p < 0.001), Stroop dots (r = 0.39, p < 0.001). The correlation between TRAIL and WRT remained significant in NC (r = 0.623, p = 0.0002), whereas, in the MCInp, the correlation was marginally significant (p = 0.06). Also, one individual with AD had a TRAIL score of 800 ms; that is, 5 standard deviations below the average score of AD on that test. This poor performance was not explained by visual impairment and was therefore treated as an outlier. Taking that subject out, revealed a significant positive correlation (p = 0.0002). The correlation between STROOP dots and WRT remained significant in the NC and MCInp. Table 2 summarises the correlation coefficients for each tests with WRT in each groups.

**Table 2:** Correlations between WRT and neuropsychological tests

		**NC**	**MCIp**	**MCInp**	**AD**
MMSE	−0.44*	−0.05	−0.26	−0.22	−0.57*
LM2	−0.25	−0.13	−0.03	−0.09	0.25
VR2	−0.32*	−0.35	−0.01	−0.02	0.15
RAVLT delayed	−0.35*	−0.16	−0.17	−0.01	0.29
COWA lexical	−0.35*	−0.53*	0.00	−0.20	−0.04
COWA semantic	−0.34*	−0.39	0.20	−0.16	0.26
BNT	−0.32*	0.05	0.01	−0.44	0.15
TRAIL	0.48*	0.62*	0.42†	0.02	0.66*
STROOP dot	0.39*	0.43*	0.48*	−0.32	0.5
STROOP word	0.27	0.34	0.56*	0.08	−0.13
STROOP color	0.22	0.30	0.40	0.25	−0.20

These numbers represent pearson correlations. In the first column, are the partial correlations of all the subjects, controlling for education and depression. * marks significant correlations (p < 0.005) and † indicates a marginal correlation (p = 0.006).

## Discussion

Similar to previous findings from this laboratory [15], we found a significant difference in the WRT scores of the AD compared to the MCI and NC groups. Even though the WRT classified correctly NC and AD 81% of the time, it showed lower diagnostic sensitivity (72%) and specificity (76%) than previously found (77% and 92% respectively). On average, the AD subjects included in our study have shorter WRT and the MCI have somewhat longer WRT than those in Massoud’s study (Massoud: MCI 60.3 ± 15.9 ms and AD 122.6 ± 70.8 ms; Present study: MCI 73.4 ± 25.7 ms and AD 92.6 ± 31.7 ms).

It is worth noting that we found slightly bigger standard deviations in the NC and MCI groups, and smaller ones in the AD group than what was previously reported, indicating that our groups might have been less homogenous. Examining the individuals scores from Massoud’s study [15] and our own replication study illustrates this difference clearly (Figure 2). In the pilot study, only 1 out of 12 NC had a WRT score greater than the 85 ms, compared to 8 out of 33 in our follow-up study. Similarly, in Massoud’s study, only 3 out of 13 AD patients scored below the cut-off, compared with 8 out of 25 in the current study. We simply failed to maintain this striking separation between NC and AD patients in a larger cohort. Even though the age and dementia severity, measured by MMSE, of the groups were similar in the two studies, we must conclude that the pilot study, being smaller, simply showed less heterogeneity than was found in our replication study.

The ROC analyses showed that WRT does not perform better than chance level in classifying MCI individuals who will progress to AD or not (AUC = 0.45, p > 0.1). Indeed, in the current study, WRT only correctly classified 32% of the MCI individuals who progressed to dementia, which is less impressive than what Massoud and his colleagues had found, where all of the MCI subjects who had scored ≥ 85 ms on WRT had progressed to AD after 2 years of follow-up. It is important to note that the follow-up period in our study was of 8 years. Massoud’s study included 4 individuals with MCI who progressed to AD, which is within the expected published rates of progression to AD (15% a year). The current study, in contrast, yielded a larger sample of converters. However, the average rate of progression from MCI to AD in our study (about 4% a year) is lower than in most studies [17]. In addition, examination of the AUC for other neuropsychological tests reveals that the delayed recall of Logical Memory and Ray Auditory Verbal Learning Tests were better at discriminating MCIp from MCInp (Table 1).

Massoud and colleagues found that WRT scores correlated with scores on a global cognitive assessment test, the MMSE. In our study, we replicated this finding, but we wanted to evaluate whether WRT scores were related to those of other cognitive tests. We found evidence for an association between WRT scores and measures of memory and language. These associations might be spurious, however, as they were found only when the three groups were collapsed, with the exception of the lexical subtest of the Controlled Word Association test in the NC group. WRT scores were also correlated with measures of attention, more precisely Trail Making Test A and Stroop dots subtest. This suggests that performance on WRT is related to overall mental speed and attention processing. However, we found that the association with Trail Making Test A was maintained in the NC, MCInp, and AD groups, but not in the MCIp group. Similarly, the association with Stroop dots remained significant only in the NC and MCInp groups. Although explaining the absence of correlation in the MCIp or AD groups is beyond the scope of this study, it might suggest that at the MCI stage of AD pathology different cognitive domains are affected to different degrees. As for the absence of correlation between MMSE and WRT scores in the NC and MCI groups, it can easily be explained by ceiling effect. In the NC and MCI groups, MMSE scores do not vary enough, whereas in the AD group, MMSE scores range from 11 to 29.

Despite the fact that we selected the participants of our study in the same way as Massoud and colleagues did, MCI individuals whose WRT was greater than 85 ms were not more likely to progress to AD after 8 years follow-up. This can be explained again by the larger sample size. The original study was a small pilot study and it is common for larger samples to lack the robust separation found in smaller, more homogeneous samples. Without doubt, our larger sample introduced heterogeneity, as expressed in the increased standard deviations in WRT scores of NC and MCI individuals. The follow up period in our replication study was significantly longer than in Massoud’s study, which brings more support to the present findings. The participants included in our replication study compared fairly well to those of Massoud’s. They had similar education and MMSE score, as well as similar duration of illness (this study: 3.9 (0–15), Massoud’s study: 4.5 (2–10) for the MCI and AD groups. The participants also compared well in term of age and BNT scores.

## Conclusions

As is the case with many studies that have looked for simple tests discriminating between MCI individuals who will progress to AD and those who will not, the results are not sufficiently robust to suggest WRT as a prognostic test. Apart from obvious methodological differences in diagnosing MCI, these disappointing results might be explained by the now established heterogeneity of AD and the increasingly recognized heterogeneity of MCI. For example, some patients with AD will show greater difficulties with language or executive functions, whereas others will show greater visuoperceptual deficits, this, aside from the frequent comorbidity of other dementia [25,26]. This heterogeneity of symptoms is also portrayed in the subdivisions of MCI; that is, pure amnestic MCI, multiple-domains (including memory) MCI, single non-memory domain MCI, and multiple non-memory domains MCI. The use of a panel of simple neuropsychological tests, in conjunction with imaging and possibly genetic biomarkers remains potentially more valuable in predicting progression to AD Petersen, [27].

## Competing interests

The authors declare that they have no competing interests.

## Authors’ contributions

GAL performed the statistical analyses, analysed the data, interpreted the results, and drafted the manuscript. HB helped with interpretation of the results, and edited the manuscript. HC designed the study, helped with interpretation of the results, and edited the manuscript. All authors read and approved the final manuscript.

## Pre-publication history

The pre-publication history for this paper can be accessed here:

http://www.biomedcentral.com/1471-2318/12/38/prepub
